# Liver stiffness measured by magnetic resonance elastography in early recurrence of hepatocellular carcinoma after treatment: A protocol for systematic review and meta analysis

**DOI:** 10.1097/MD.0000000000026183

**Published:** 2021-06-11

**Authors:** Huiyan Zhao, Lijun Zhang, Huadong Chen

**Affiliations:** Department of Radiological, The Central Hospital of Enshi Tujia and Miao Autonomous Prefecture, Enshi, Hubei Province, China.

**Keywords:** diagnosis, hepatocellular carcinoma, liver stiffness, magnetic resonance elastography, meta-analysis, protocol, recurrence

## Abstract

**Background:**

With high diagnostic accuracy, magnetic resonance elastography (MRE) is a noninvasive tool and can be adopted to measure liver stiffness (LS). In this study, meta-analysis was carried out to further evaluate whether LS measured by MRE can predict early recurrence in patients with hepatocellular carcinoma (HCC).

**Methods:**

PUBMED, EMBASE, Web of Science, China National Knowledge Infrastructure, and Cochrane Library database were searched for studies related to LS measured by MRE in the prediction of recurrence in patients with HCC. Survival outcome was estimated by hazard ratios and 95% confidence intervals. Meta-analysis was conducted with the Stata 16.0.

**Results:**

The results of this meta-analysis will be submitted to a peer-reviewed journal for publication.

**Conclusion:**

This study will provide evidence support for LS measured by MRE in predicting the recurrence of HCC.

**Ethics and dissemination:**

The private information from individuals will not be published. This systematic review also should not damage participants’ rights. Ethical approval is not available. The results may be published in a peer-reviewed journal or disseminated in relevant conferences.

**OSF Registration Number:**

DOI 10.17605/ OSF.IO / SURH3.

## 1 Introduction

Hepatocellular carcinoma (HCC) is the fifth most common cancer in the world and the third leading cause of cancer-related death.^[1,2]^ The treatment of HCC includes liver transplantation, microwave ablation, radiofrequency ablation, and hepatectomy.^[3–5]^ Although these treatments improve the therapeutic effects of HCC, recurrence is still common after treatment, thus resulting in a low survival rate.^[6,7]^

Magnetic resonance elastography (MRE), known as “‘image palpation,” is a new noninvasive imaging technique.^[8,9]^ Mechanical waves can be used to quantitatively quantify the degree of liver fibrosis, fat content and iron content. The comprehensiveness and accuracy of its imaging are better than those of ultrasound-based elastography such as shear wave elastography and instantaneous elastography. At present, MRE is used for the examination of many organs in the whole body, including liver, heart, brain, and so on. The application of MRE in the examination of liver diseases is quite mature.^[10]^ Liver stiffness (LS) is closely correlated with the degree of liver fibrosis.^[11]^ Accurate staging of liver fibrosis is very important in determining the treatment plan and follow-up interval of patients suffering from chronic liver disease.^[12]^ LS measured by MRE may be a predictor of early recurrence of HCC after treatment.

Although some recent studies have reported that LS measured by MRE can be regarded as a predictor of early recurrence after HCC treatment, the sample size of these studies is limited.^[13–17]^ In this study, we searched all relevant studies to systematically evaluate the value of LS measured by MRE as a predictor of early recurrence after HCC treatment.

## 2 Methods

### 2.1 Study registration

The protocol of the systematic review has been registered on Open Science Framework (registration number: DOI 10.17605 / OSF.IO / SURH3). It was reported by following the guideline of Preferred Reporting Items for Systematic Reviews and Meta-analysis Protocol Statement.^[18]^

### 2.2 Inclusion criteria for study selection

We included studies that met the following criteria:

1. Patients with HCC under the Milan criteria and who underwent hepatic resection, radiofrequency ablation, or transarterial chemoembolization as primary treatment.
2. The relationship between LS measured by MRE and the prognosis of HCC patients was analyzed. Prognostic indicators are recurrence-free survival.
3. Sufficient data were included to extract or calculate the hazard ratio (HR).

Exclusion criteria:

1. Insufficient data were extracted or calculated for HR and its 95% confidence interval (CI).
2. Literatures with repeated research data.
3. Case reports, reviews, cell, or animal studies.

### 2.3 Data sources and search strategy

PUBMED, EMBASE, Web of Science, China National Knowledge Infrastructure, and Cochrane Library database were searched for related literatures in Chinese or English regarding literatures involved LS measured by MRE predict early recurrence in HCC patients. The publication time was from inception to May 2021. The search strategy for PubMed is displayed in Table 1.

**Table 1:** PubMed search strategy.

Number	Search terms
#1	Carcinoma, Hepatocellular[MeSH]
#2	Hepatocellular Carcinoma[Title/Abstract]
#3	Hepatoma[Title/Abstract]
#4	Liver Cancer, Adult[Title/Abstract]
#5	Liver Cell Carcinoma[Title/Abstract]
#6	Liver Cell Carcinoma, Adult[Title/Abstract]
#7	Adult Liver Cancer[Title/Abstract]
#8	Adult Liver Cancers[Title/Abstract]
#9	Cancer, Adult Liver[Title/Abstract]
#10	Cancers, Adult Liver[Title/Abstract]
#11	Carcinoma, Liver Cell[Title/Abstract]
#12	Carcinomas, Hepatocellular[Title/Abstract]
#13	Carcinomas, Liver Cell[Title/Abstract]
#14	Cell Carcinoma, Liver[Title/Abstract]
#15	Cell Carcinomas, Liver[Title/Abstract]
#16	Hepatocellular Carcinomas[Title/Abstract]
#17	Hepatomas[Title/Abstract]
#18	Liver Cancers, Adult[Title/Abstract]
#19	Liver Cell Carcinomas[Title/Abstract]
#20	or/1–19
#21	Elasticity Imaging Techniques[MeSH]
#22	Magnetic resonance elastography[Title/Abstract]
#23	ARFI Imaging[Title/Abstract]
#24	Acoustic Radiation Force Impulse Imaging[Title/Abstract]
#25	Elastograms[Title/Abstract]
#26	Elastography[Title/Abstract]
#27	Magnetic Resonance Elastography[Title/Abstract]
#28	Sonoelastography[Title/Abstract]
#29	Tissue Elasticity Imaging[Title/Abstract]
#30	Vibro-Acoustography[Title/Abstract]
#31	ARFI Imagings[Title/Abstract]
#32	Elasticity Imaging Technique[Title/Abstract]
#33	Elasticity Imaging, Tissue[Title/Abstract]
#34	Elasticity Imagings, Tissue[Title/Abstract]
#35	Elastogram[Title/Abstract]
#36	Elastographies[Title/Abstract]
#37	Elastographies, Magnetic Resonance[Title/Abstract]
#38	Elastography, Magnetic Resonance[Title/Abstract]
#39	Imaging Technique, Elasticity[Title/Abstract]
#40	Imaging Techniques, Elasticity[Title/Abstract]
#41	Imaging, ARFI[Title/Abstract]
#42	Imaging, Tissue Elasticity[Title/Abstract]
#43	Imagings, ARFI[Title/Abstract]
#44	Imagings, Tissue Elasticity[Title/Abstract]
#45	Magnetic Resonance Elastographies[Title/Abstract]
#46	Resonance Elastographies, Magnetic[Title/Abstract]
#47	Resonance Elastography, Magnetic[Title/Abstract]
#48	Sonoelastographies[Title/Abstract]
#49	Technique, Elasticity Imaging[Title/Abstract]
#50	Techniques, Elasticity Imaging[Title/Abstract]
#51	Tissue Elasticity Imagings[Title/Abstract]
#52	Vibro Acoustography[Title/Abstract]
#53	Vibro-Acoustographies[Title/Abstract]
#54	or/21–53
#55	Liver stiffness[Title/Abstract]
#56	Recurrence[MeSH]
#57	Recrudescence[Title/Abstract]
#58	Relapse[Title/Abstract]
#59	Recrudescences[Title/Abstract]
#60	Recurrences[Title/Abstract]
#61	Relapses[Title/Abstract]
#62	or/56–61
#63	Prognos^∗^[Title/Abstract]
#64	Survival [Title/Abstract]
#65	or/63–64
#66	#20 and #54 and #55 and #62 and #65

### 2.4 Data collection and analysis

#### 2.4.1 Study selection

**Figure 1:**
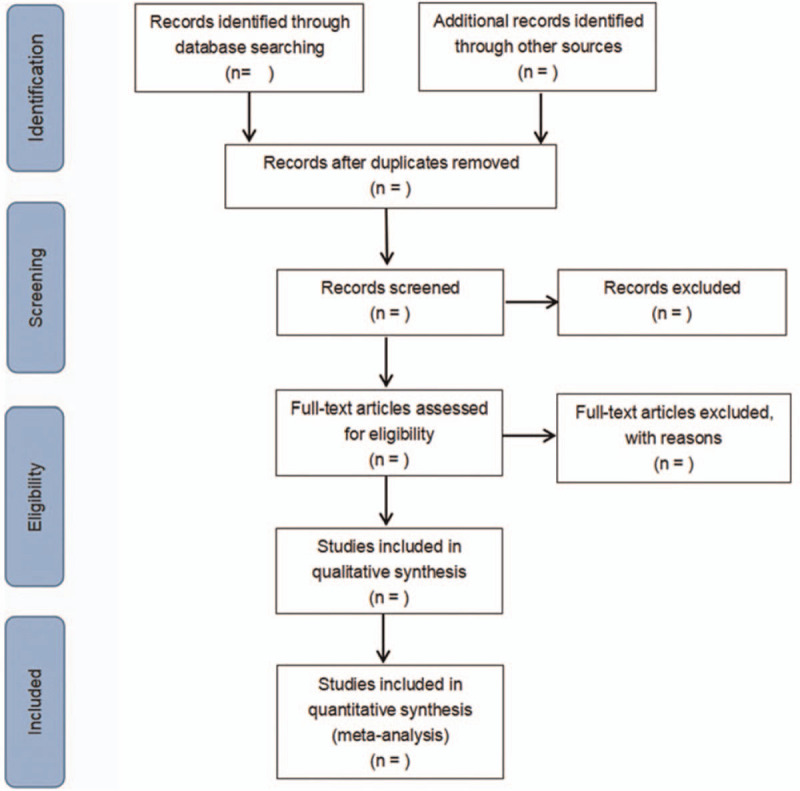
Flow diagram of literature retrieval.

The screening flow chart of this study is demonstrated in Figure 1. According to the established inclusion and exclusion criteria, 2 researchers independently screened the literature and extracted the information and cross-checked. If there exists any inconsistency, it will be resolved through negotiation.

#### 2.4.2 Data extraction

The extracted information mainly includes the first author, publication year, country, study type, surgery type, threshold, sample size, the longest follow-up period, univariate analysis, multivariate analysis, etc. Furthermore, in view of the fact that some studies only provide Kaplan–Meier curves, it is necessary to apply Engauge Digitizer4.1 version to extract HR and its 95% CI from graphic survival curves.^[19,20]^

#### 2.4.3 Dealing with missing data

If there are insufficient or missing data in the literature, the authors will be contacted via email. If the data are still not available, only the current available data will be analyzed and the potential impacts will be discussed.

### 2.5 Quality assessment

The quality of the included references was assessed using the Newcastle-Ottawa Scale (NOS).^[21]^ Score ≥ 7 indicates that the quality of the literature is high.^[22]^

### 2.6 Statistical analysis

All of the above statistical analyses were performed with Stata 16.0 (StataCorp LLC, college station, TX). Recurrence-free survival was taken as prognostic outcomes, and the results were expressed as HRs with 95% CIs. Heterogeneity was tested by Q-statistic and *I*^2^-statistic, *I*^2^ > 50% was considered as significant heterogeneity, and the random-effects model or the fixed-effects model was adopted. *P* values in this study were two-sided, and *P* < .05 suggested that there was a statistical significance.

### 2.7 Subgroup analysis

A subgroup analysis will be made on the basis of study type, surgery type, and threshold.

### 2.8 Sensitivity analysis

We will adopt the one-by-one exclusion method to analyze the sensitivity of the results.

### 2.9 Reporting bias

Egger linear regression test and Begg rank test were conducted to assess publication bias.^[23,24]^

### 2.10 Ethics and dissemination

Since the program does not include the recruitment of patients and the collection of personal information, it does not require the approval of the Ethics Committee.

## 3 Discussion

MRE is the most accurate technique for non-invasive staging of hepatic fibrosis.^[25]^ At present, MRE measurement of LS has been considered as a potential biomarker, and it can objectively quantify liver fibrosis to predict the future prognosis of patients suffering from chronic liver disease.^[26–28]^ A recent study revealed that MRE may be a potential biomarker for predicting the future risk of HCC development in patients with chronic liver disease.^[29]^ In addition, LS measured by MRE has many advantages and can be potentially used as a biomarker of recurrence in patients with HCC. In this study, meta-analysis was performed to evaluate the LS prediction measured by MRE as a predictor of HCC recurrence, which will provide evidence for early recurrence treatment of HCC.

## Author contributions

**Conceptualization:** Huadong Chen, Huiyan Zhao, Lijun Zhang.

**Data curation:** Huadong Chen, Huiyan Zhao, Lijun Zhang.

**Funding acquisition:** Huadong Chen.

**Project administration:** Huadong Chen.

**Resources:** Huadong Chen.

**Software:** Huiyan Zhao.

**Supervision:** Huiyan Zhao.

**Validation:** Lijun Zhang.

**Visualization:** Lijun Zhang.

**Writing – original draft:** Huadong Chen, Huiyan Zhao, Lijun Zhang.

**Writing – review & editing:** Huadong Chen, Huiyan Zhao, Lijun Zhang.
